# Downregulation of microRNA-30a-5p contributes to the replication of duck enteritis virus by regulating Beclin-1-mediated autophagy

**DOI:** 10.1186/s12985-019-1250-5

**Published:** 2019-11-26

**Authors:** Xianglong Wu, Renyong Jia, Mingshu Wang, Shun Chen, Mafeng Liu, Dekang Zhu, Xinxin Zhao, Qiao Yang, Ying Wu, Zhongqiong Yin, Shaqiu Zhang, Juan Huang, Ling Zhang, Yunya Liu, Yanling Yu, Leichang Pan, Bin Tian, Mujeeb Ur Rehman, Xiaoyue Chen, Anchun Cheng

**Affiliations:** 1 Research Center of Avian Disease, College of Veterinary Medicine of Sichuan Agricultural University, Wenjiang District, Chengdu, 611130 Sichuan Province China; 2 Institute of Preventive Veterinary Medicine, Sichuan Agricultural University, Wenjiang District, Chengdu, 611130 Sichuan Province China; 3 Key Laboratory of Animal Disease and Human Health of Sichuan Province, Wenjiang District, Chengdu, 611130 Sichuan Province China

**Keywords:** Duck enteritis virus, Autophagy, miR-30a-5p, Beclin-1

## Abstract

**Background:**

MicroRNAs (miRNAs) is increasingly recognized as an important element in regulating virus-host interactions. Our previous results showed that cellular miR-30a-5p was significantly downregulated after duck enteritis virus (DEV) infection cell. However, whehter or not the miR-30a-5p is involved in DEV infection has not been known.

**Methods:**

Quantitative reverse-transcription PCR (qRT-PCR) was used to measure the expression levels of miRNAs(miR-30a-5p) and Beclin-1 mRNA. The miR-30a-5p - Beclin-1 target interactions were determined by Dual luciferase reporter assay (DLRA). Western blotting was utilized to analyze Beclin-1-mediated duck embryo fibroblast (DEF) cells autophagy activity. DEV titers were estimated by the median tissue culture infective dose (TCID_50_).

**Results:**

The miR-30a-5p was significantly downregulated and the Beclin-1 mRNA was significantly upregulated in DEV-infected DEF cells. DLRA confirmed that miR-30a-5p directly targeted the 3′- UTR of the Beclin-1 gene. Overexpression of miR-30a-5p significantly reduced the expression level of Beclin-1protein (*p* < 0.05), leading to the decrease of Beclin-1-mediated autophagy activity, which ultimately suppressed DEV replication (*P* < 0.05). Whereas transfection of miR-30a-5p inhibitor increased Beclin-1-mediated autophagy and triggered DEV replication during the whole process of DEV infection (*P* < 0.01).

**Conclusions:**

This study shows that miR-30a-5p can inhibit DEV replication through reducing autophagy by targeting Beclin-1. These findings suggest a new insight into virus-host interaction during DEV infection and provide a potential new antiviral therapeutic strategy against DEV infection.

## Background

Duck enteritis virus (DEV) is the causative pathogen of duck viral enteritis disease, causing considerable economic losses in the duck industry due to high mortality and low egg production [1, 2]. In addition, DEV can cause variable morbidity and mortality in geese, swans and other wild waterfowl and poses a severe threat to waterfowl groups [3–5]. DEV is classified into the family *Herpesviridae*, subfamily *Alphaherpesvirinae*, genus *Mardivirus* and *anatid herpesvirus I* [6]. Its genome is a linear double-stranded DNA molecule that is composed of a unique long region (UL) and a unique short region (US) flanked by a short internal repeat sequence (IRS) and a short terminal repeat sequence (TRS) [7–10].

Autophagy is an essential self-digestion process that degrades protein and waste in cells to maintain cellular metabolic balance and homeostasis [11, 12]. Growing evidence has shown that viral infection can induce cellular autophagy. For example, some viruses, such as Newcastle disease virus (NDV) [13], classical swine fever virus (CSFV) [14], porcine circovirus type 2 (PCV2) [15], porcine reproductive and respiratory syndrome virus (PRRSV) [16], dengue virus, foot-and-mouth disease virus (FMDV) and varicella-zoster virus (VZV) [17], can induce cell autophagy to enhance their replication. However, autophagy can suppress viral replication and eliminate viral infection [18, 19]. For example, cellular autophagy can inhibit replication of vesicular stomatitis virus (VSV) by regulating the P13K/AKT signaling pathway [20]. Recent research has reported that DEV induces autophagy to enhance its replication in duck embryo fibroblast (DEF) cells [21]. Nevertheless, the regulatory relationship of autophagy remains poorly understand.

MicroRNAs (miRNAs) are important small (18–24 nt), noncoding, endogenous RNAs that can negatively regulate gene expression by binding fully or partially to the 3′-untranslated region (3′-UTR) [22, 23]. Accumulating evidence has demonstrated that miRNAs participate in a wide range of biological processes, including cellular proliferation, differentiation, signal transduction, metabolism apoptosis and cellular autophagy [24–27], and play important roles in regulating virus-host interactions [28–30]. Our previous high-throughput sequencing results revealed that 13 cellular miRNAs (mir-125-2-3p, mir-124a-3p, mir-215-5p, mir-29b-3p, etc) were significantly upregulated and 25 miRNAs (mir-1a-3p, mir-133a-5p, miR-30a-5p, miR-16c-5p, etc) were significantly downregulated after CHv infection [31]. Therefore, we speculate that these miRNAs may play crucial roles in DEV infection.

In this study, we first confirmed that miR-30a-5p directly targeted the 3′-UTR of the Beclin-1 mRNA. Further study showed that overexpression of miR-30a-5p inhibited DEV replication by downregulating Beclin-1-mediated autophagy in DEF cells. miR-30a-5p inhibitor triggered DEV replication, suggesting that miR-30a-5p palys important roles in the regulation of DEV-induced autophagy and viral proliferation. These data provide a basis for further understanding miRNAs’ regulatory roles in cellular autophagy and should contribute to the development of anti-DEV drugs.

## Methods

### Virus, cells, miRNA mimic and antibodies

The DEV CHv (Chinese virulent strain) (accession No. JQ647509) and mouse anti-UL41 serum were provided by the Avian Diseases Research Center, College of Veterinary Medicine, Sichuan Agricultural University. Duck embryo fibroblast (DEF) cultures were prepared from 10-day-old duck embryos for the propagation of CHv. The study was approved by the Animal Ethics Committee of Sichuan Agricultural University (approval No. XF2016–17). Cell monolayers were cultured in Dulbecco’s Modified Eagle’s Medium (DMEM, Gibco, Grand Island, NY USA) supplemented with 10% fetal bovine serum (FBS, Gibco, USA) and 1% penicillin-streptomycin (Gibco, USA) at 37 °C in a 5% CO_2_ atmosphere. The miR-30a-5p mimic, mimic negative-control (mimic-NC), miR-30a-5p inhibitor and inhibitor-NC were synthesized by Ribobio (Guangzhou, China) and transfected into cells at a final concentration of 100 nM.

### Quantitative real-time RT-PCR

Stem-loop qRT-PCR and general qRT-PCR methods were used to measure the expression levels of miRNAs and Beclin-1 mRNA, respectively. Total RNA from DEV-infected and uninfected DEF cells was extracted with TRIzol reagent (TIANGEN Biotech, Beijing) and quantified using a spectrophotometer (NanoDrop 2000). RNA (1000 ng) was reverse-transcribed to cDNA, and then 2 μl cDNA was used for real-time PCR amplification according to the kit manufacturer’s (Thermo) instructions. The primers are listed in Table 1. Relative expression levels of miRNA and Beclin-1 mRNA were calculated using the 2^-ΔΔCt^ method. U6 and β-actin were used as respective endogenous controls.

**Table 1:** Primers for analysis of gene expression by qRT-PCR

Primers	Sequence
RT-miR-146b-5p	GTCGTATCCAGTGCGTGTCGTGGAGTCGGCAATTGCACTGGATACGACAACGCCTA
RT-miR-125b-5p	GTCGTATCCAGTGCGTGTCGTGGAGTCGGCAATTGCACTGGATACGACCACAAGTT
RT-miR-30a-5p	GTCGTATCCAGTGCGTGTCGTGGAGTCGGCAATTGCACTGGATACGACAGCTTCCA
RT-miR-27b-3p	GTCGTATCCAGTGCGTGTCGTGGAGTCGGCAATTGCACTGGATACGACGCAGAACT
RT-miR-16c-5p	GTCGTATCCAGTGCGTGTCGTGGAGTCGGCAATTGCACTGGATACGACCTCCAGTA
RT-miR-130b-3p	GTCGTATCCAGTGCGTGTCGTGGAGTCGGCAATTGCACTGGATACGACACGCCCTT
miR-146b-5p (F)	GCCGTGAGAACTGAATTCCATA
miR-125b-5p (F)	GCCGTCCCTGAGACCCTAA
miR-30a-5p (F)	GCCGTGTAAACATCCTTGACTG
miR-27b-3p (F)	GCCGTTCACAGTGGCTAAG
miR-16c-5p (F)	GCCGTAGCAGCACGTAAATA
miR-130b-3p (F)	GCCGCAGTGCAATAATGAAA
UR-primer	CAGTGCGTGTCGTGGAGT
U6 (F)	CTCGCTTCGGCAGCACA
U6 (R)	GCGTGTCATCCTTGCGC
Beclin-1 (F)	AAGAGGTGCCTGGAGATCCT
Beclin-1 (R)	CGTCCTCCAGCTCCTGAATC
β-Actin (F)	CCGGGCATCGCTGACA
β-Actin (R)	GGATTCATCATACTCCTGCTTTGCT

### Vector constructs and luciferase assay

MiR-30a-5p was predicted to target the DEF Beclin-1 3’UTR (nt 136,000-145,890) according to RNAhybrid and PITA software. The Beclin-1 3’UTR (nt 136,085-136,248), including the predicted miR-30a-5p binding site, was synthesized by TsingKe (Chengdu, China) and cloned into a pmirGLO vector (Promega, Madison, WI, USA) with SacI and XhoI sites, resulting in pmirGLO-WT-Beclin-1. Accordingly, the mutant 3’UTR of the Beclin-1 vector was constructed and named pmirGLO-MU-Beclin-1. For the luciferase assay, COS7 cells were seeded in 96-well plates and cotransfected with miR-30a-5p mimic, miR-NC, pmirGLO-WT-Beclin-1 and pmirGLO-MU-Beclin-1 with Lipofectamine 3000 (Invitrogen, Carlsbad, CA, USA). We performed a site-directed DLRA, and luciferase activity was measured at 36 h posttransfection according to the manufacturer’s protocol (Promega, Madison, WI, USA).

### Western blot analysis

The synthetic miR-30a-5p mimic, miR-NC, miR-30a-5p inhibitor and inhibitor-NC were transfected into DEF cells with Lipofectamine 3000 (Invitrogen) according to the manufacturer’s protocol. Meanwhile, the blank group (without) was set as the control group. Cells were infected with DEV at a multiplicity of infection (MOI) of 1.0 for 36 h. The cells were harvested and washed 3 times with cold PBS. The PBS was decanted and then 150 μl RIPA lysis buffer (Solarbio, China) and 1.0 mM PSMF were added. After 30 min on ice and centrifugation at 12,000 g for 10 min, 25 μl supernatant was mixed with 25 μl 5 × SDS loading buffer and boiled for 10 min. The protein samples were analyzed by 12% SDS polyacrylamide gel electrophoresis and transferred to a polyvinylidene difluorride (PVDF) membranes (Millipore, Billerica, MA) by electroelution. Membranes were blocked with 5% milk-TBS-Tween-20 for 2 h at room temperature and incubated with rabbit anti-LC3 (Proteintech, 14,600–1-AP), rabbit anti-p62/SQSTM1 (Cell Signaling Technology, 5114), mouse anti-β-actin (Proteintech, 60,008–1-Ig), rabbit anti-Beclin-1 (Proteintech, 11,306–1-AP) and anti-CHv (UL41) antibodies overnight at 4 °C. Following incubation with HRP-conjugated goat anti-rabbit or anti-mouse IgG (Biodragon-Immunotech, China) as secondary antibody for 2 h at 37 °C, the immunoreactive bands were detected using an enhanced chemiluminescence kit (Solarbio, China). The amount of proteins was quantified by densitometry and normalized to β-actin, an internal standard.

### Flow cytometry assay

DEF cells were seed in a 6-well plate at adensity of 1 × 10^6^ cells per well. Cells were pretreated with control (without), miR-30a-5p mimic, miR-NC, miR-30a-5p inhibitor and inhibitor-NC for 4 h and then infected with DEV (MOI = 1.0) for 36 h. The cells were stained with Annexin V-fluorescein isothiocyanate (V-FITC) (BD Pharmingen, USA) and propidium iodide (PI) (BD Pharmingen, USA) according to the manufacturer’s instructions, and the percentage of apoptotic cells was assayed by flow cytometry (FCM).

### Cell viability analysis

miRNA toxicity tests were performed using the MTT assay kit (Sangon Biotech, Shanghai, China) according to the manufacturer’s instructions. In brief, DEF cells were seeded in 96-well culture plates at a density of 1 × 10^5^ cells per well. Cells were pretreated with control, miR-30a-5p mimic, miR-NC, miR-30a-5p inhibitor and inhibitor-NC for 4 h and then cultured in DMEM for 36 h, the cells were incubated in 100 μl fresh culture medium containing MTT (0.5 mg mL^− 1^) for 4 h at 37 °C. The medium was replaced by 100 μl formazan solubilization solution, and the absorbance was measured at 570 nm using a microplate reader (Bio-Rad).

### DEV replication analysis

DEV viral copies were detected using qRT-PCR methods. DEF cells were seed in a 6-well plate at adensity of 1 × 10^6^ cells per well. Cells were pretreated with control, miR-30a-5p mimic, miR-NC, miR-30a-5p inhibitor and inhibitor-NC for 4 h and then infected with DEV (MOI = 1.0). The cells were collected at the indicated times and stored at − 80 °C for subsequent experiments. The DEV absolute quantitative curve was created as previously described methods [32, 33].

DEV titers were estimated by the median tissue culture infective dose (TCID_50_). DEF cells were seed in 96-well plates at adensity of 1 × 10^5^ cells per well. Cells were pretreated with control, miR-30a-5p mimic, miR-NC, miR-30a-5p inhibitor and inhibitor-NC for 4 h and then infected with DEV collected at the indicated times above. The plates were incubated for 5 days at 37 °C in a 5% CO_2_ atmosphere. Cell pathological changes were observed under a light microscope and recorded. Viral titers were measured according to the Reed-Muench method [34].

### Statistical analysis

Each assay was performed in three independent experiments. All experimental results are expressed as the mean ± standard deviation (mean ± SD) and were analyzed by the software GraphPad Prism (version 7.0). The statistical significance was assessed using Student’s t-test. *p < 0.05 and **p < 0.01 indicate significance.

## Results

### DEV infection induces downregulation of miR-30a-5p and promotes Beclin-1 mRNA expression

**Fig. 1:**
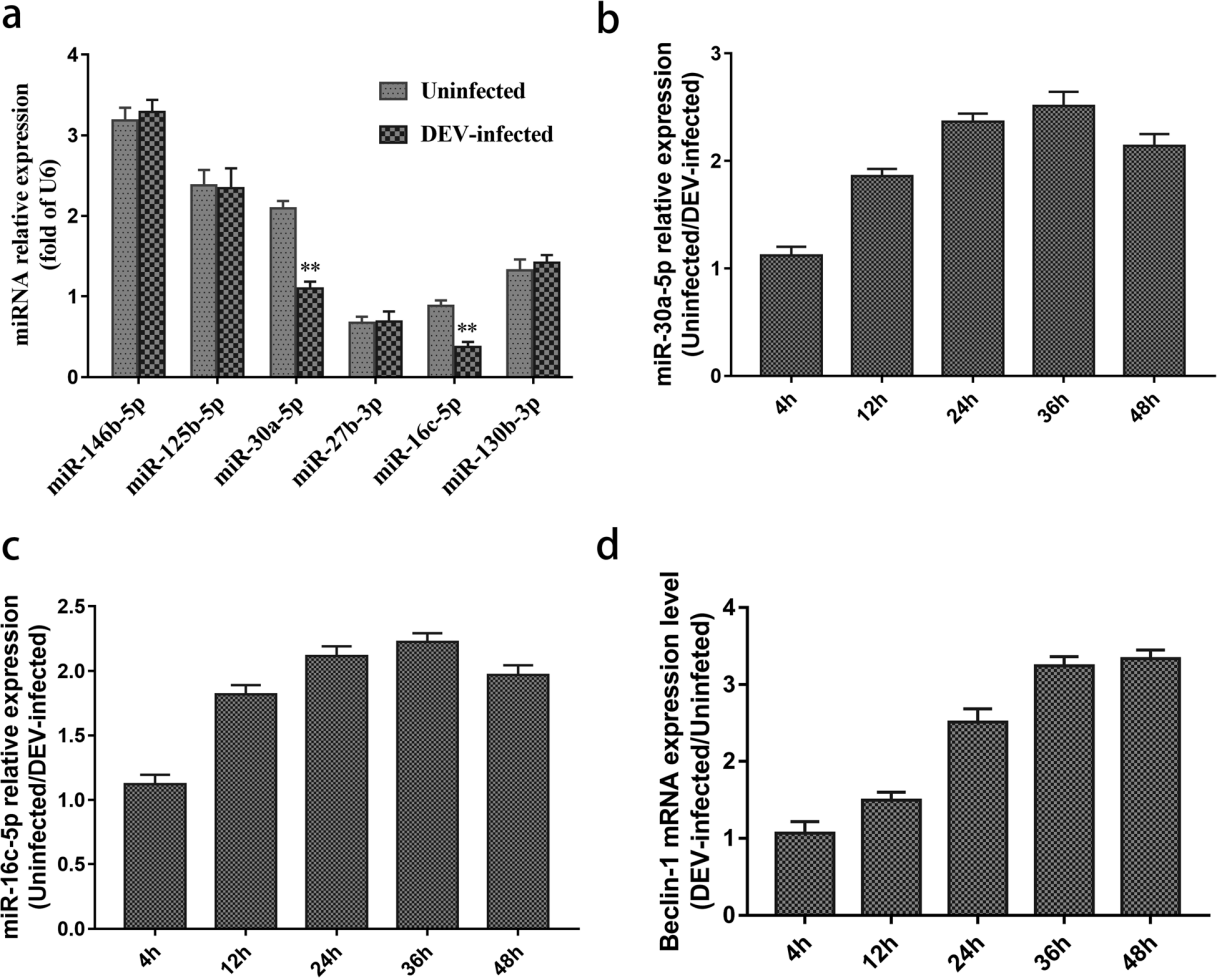
Expression levels of miRNAs and Beclin-1 mRNA after DEV infection of DEF cells. **a** Expression levels of 6 DEF-encoded miRNAs were detected using stem-loop qRT-PCR at 36 hpi. **b** Expression of miR-30a-5p was measured by stem-loop qRT-PCR at the indicated time points. U6 was used as an endogenous control. **c** Expression of miR-16c-5p was measured by stem-loop qRT-PCR at the indicated time points. U6 was used as an endogenous control. **d** Expression of Beclin-1 mRNA was measured by qRT-PCR at the indicated time points. β-Actin was used as an endogenous control. The data are presented as the mean ± SD for three independent experiments. *p < 0.05; **p < 0.01

According to our previous high-throughput sequencing results [31], six miRNAs were chosen and detected using the stem-loop qRT-PCR method. The results showed that the expression levels of miR-30a-5p and miR-16c-5p were significantly downregulated in DEV-infected DEF cells at 24 hpi (*P* < 0.01) (Fig. 1a), and there were no obvious changes in the remaining four miRNAs (miR-146b-5p, miR-27b-3p, miR-130b-3p and miR-125b-5p) compared with uninfected cells (Fig. 1a). We detected the expression levels of miR-30a-5p and miR-16c-5p at 4, 12, 24, 36 and 48 hpi in DEV-infected group and uninfected group respectively. We found that the expression levels of mir-30a-5p and mir-16c-5p in the uninfected group were 2.5 and 2.2 times as high as those in the infected group at 36 hpi respectively (Fig. 1b, c). In addition, qRT-PCR results showed that the expression level of Beclin-1 was upregulated in DEV-infected DEF cells and was 2.1- and 2.4-fold higher compared with the uninfected group at 36 hpi and 48 hpi (Fig. 1d).

### Beclin-1 is a target of miR-30a-5p in DEF cells

**Fig. 2:**
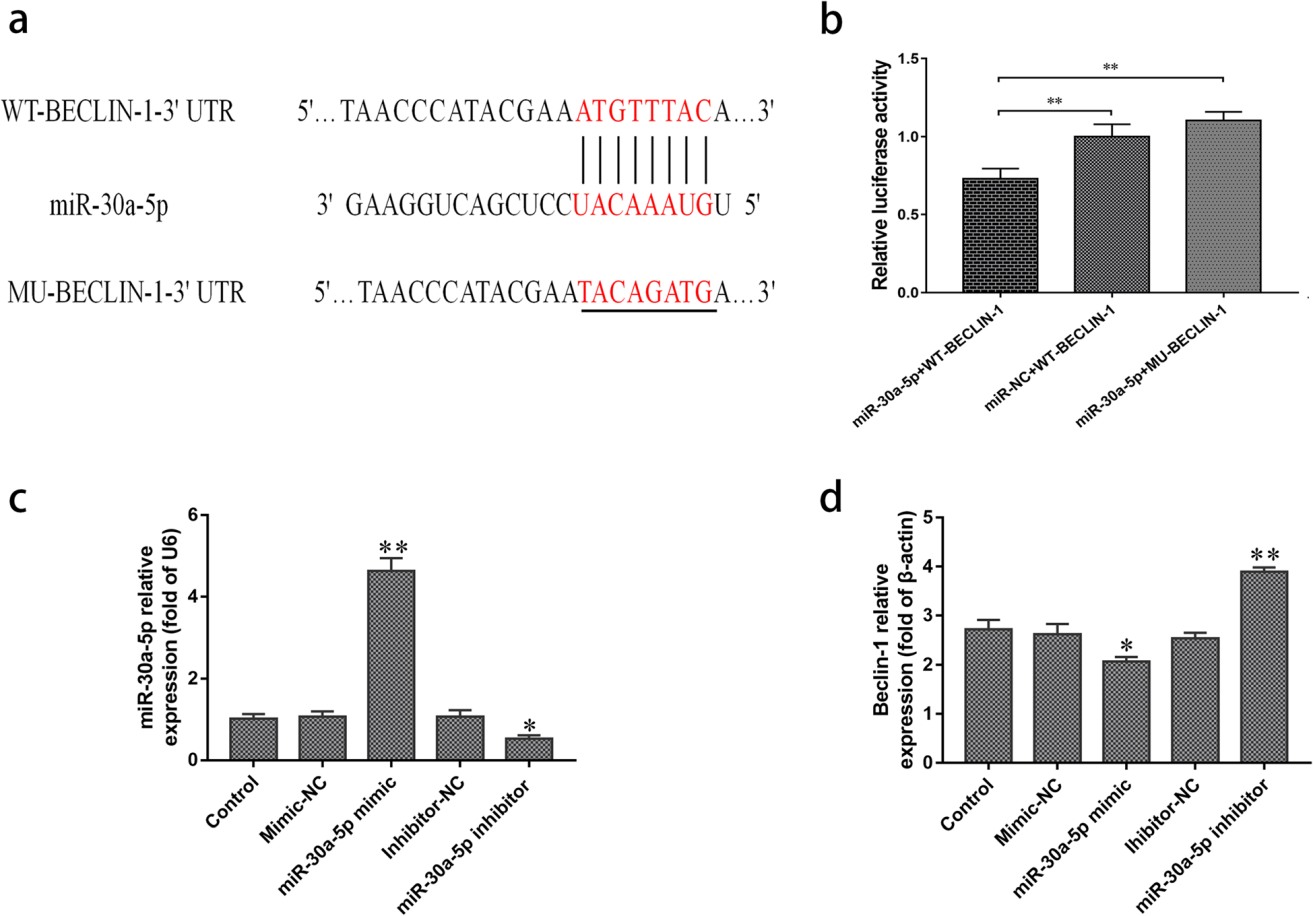
Luciferase reporter assay for the interaction between miR-30a-5p and Beclin-1 mRNA. **a** The seed sequence of miR-30a-5p and its target site in the 3’UTR of Beclin-1 mRNA are shown in red, and eight nucleotides were mutated in the 3’UTR of Beclin-1 mRNA (underlined). **b** Activity of the luciferase gene linked to the 3’UTR of Beclin-1 mRNA. Wild-type pmirGLO-WT-Beclin-1 (WT-Beclin-1) or mutant pmirGLO-MU-Beclin-1 (MU-Beclin-1) was transfected into COS7 cells with the miR-30a-5p mimic or the negative control (miR-NC). Luciferase activity was measured after 36 h. **c** DEF cells were pretreated with control (without), mimic-NC, miR-30a-5p mimic, inhibitor-NC and miR-30a-5p inhibitor for 4 h and then infected with DEV for 36 h. The expression level of miR-30a-5p was assessed by qRT-PCR. **d** DEF cells were pretreated with control (without), mimic-NC, miR-30a-5p mimic, inhibitor-NC and miR-30a-5p inhibitor for 4 h and then infected with DEV for 36 h. The expression level of Beclin-1 was assessed by qRT-PCR. The data are presented as the mean ± SD for three independent experiments. *p < 0.05; **p < 0.01, compared with the control group (miR-30a-5p + WT-Beclin-1)

A DLRA showed that pmirGLO-WT-Beclin-1 was significantly repressed by miR-30a-5p compared to the mimic-NC group (*p* < 0.01) (Fig. 2a, b). To further explore whether the downregulation of targets by miR-30a-5p was binding site-dependent, the binding sites of Beclin-1 were mutated to make the construct pmirGLO-MU-Beclin-1 vector (Fig. 2a). As expected, miR-30a-5p lost its repression effect on the mutant vector pmirGLO-MU-Beclin-1 (Fig. 2b). These results indicated that miR-30a-5p can directly target the DEF Beclin-1 mRNA at a 8-nucleotide complementary seed sequence.

### miR-30a-5p suppresses Beclin-1 expression and autophagy is induced by DEV

To further explore whether therer is the negative regulation relationship between miR-30a-5p and Beclin-1 in DEV-infected DEF cells, DEF cells were transfected with control, miR-30a-5p mimic, miR-NC, miR-30a-5p inhibitor and inhibitor-NC for 4 h and then infected with DEV (MOI = 1.0) for 36 h. The mRNA expression levels of miR-30a-5p and Beclin-1 were evaluated by qRT-PCR. As expected, Transfection of the miR-30a-5p mimic significantly increased the miR-30a-5p expression and decreased the mRNA level of Beclin-1. Consistently, miR-30a-5p inhibitor had the opposite effects on expression in miR-30a-5p and Beclin-1 (*P* < 0.01) (Fig. 2c, d).

**Fig. 3:**
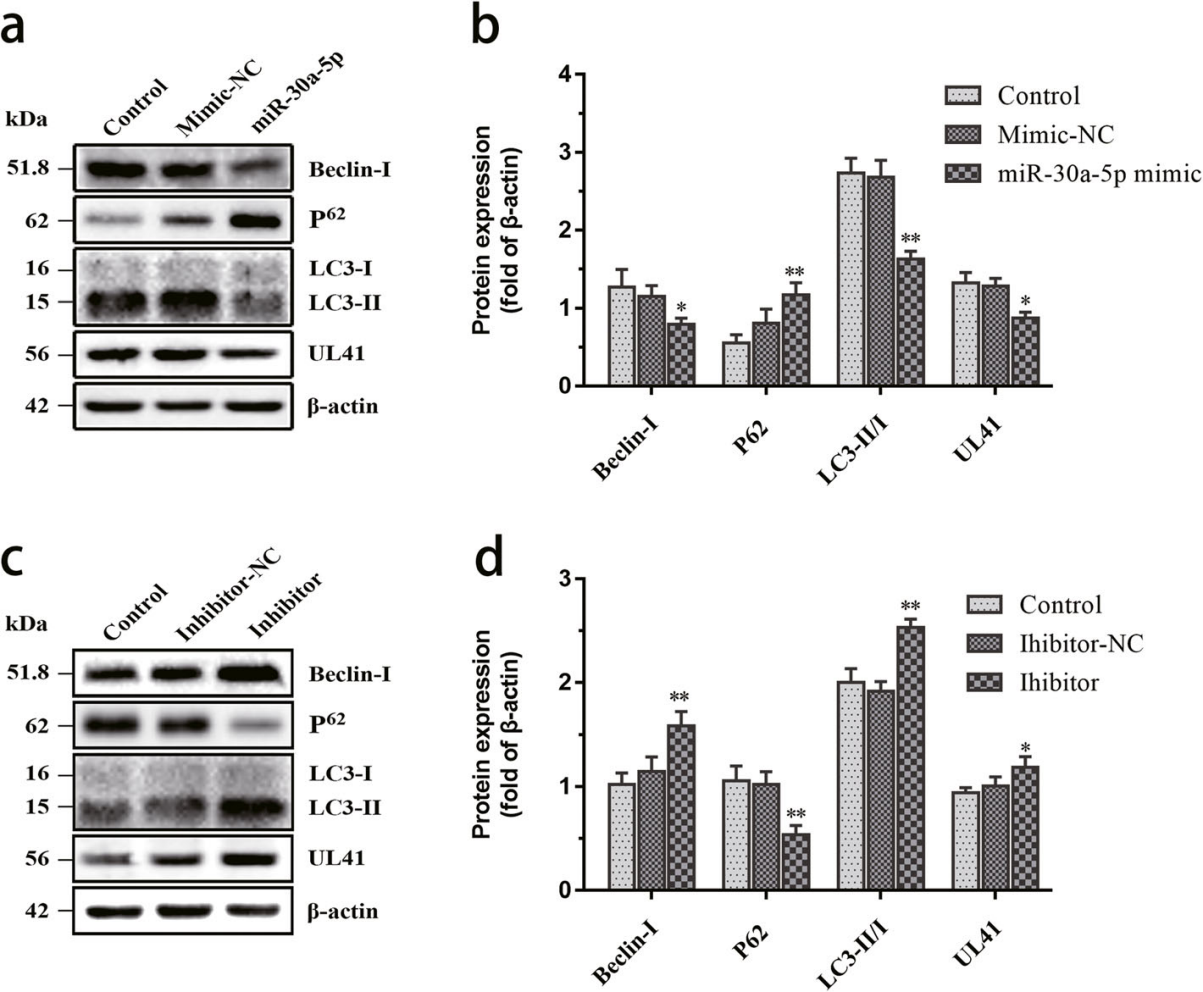
miR-30a-5p overexpression suppresses Beclin-1 expression and DEV-induced autophagy. **a** DEF cells were pretreated with control (without), mimic-NC and miR-30a-5p mimic for 4 h and then infected with DEV. Cells were lysed and blotted with antibodies against Beclin-1, p62, LC3, β-actin and UL41 at 36 hpi. **b** The optical densities of each protein band from (**a**) were measured by densitometric scanning, and the optical density ratios of Beclin-1/β-actin, P62/β-actin LC3II/I, and UL41/β-actin were calculated. **c** DEF cells were pretreated with control (without), inhibitor-NC and miR-30a-5p inhibitor for 4 h and then infected with DEV. Cells were lysed and blotted with antibodies against Beclin-1, p62, LC3, β-actin and UL41 at 36 hpi. **d** The optical densities of each protein band from (**c**) were measured by densitometric scanning, and the optical density ratios of Beclin-1/β-actin, P62/β-actin LC3II/I, and UL41/β-actin were calculated. The data are presented as the mean ± SD for three independent experiments. *p < 0.05; **p < 0.01, compared with the control group

Next, we observed the effect of miR-30a-5p on autophagy. Western blot analysis showed that miR-30a-5p overexpression significantly decreased Beclin-1 protein expression. The ratio of of LC3-II/LC3-I was significantly downregulated and the p62 protein level was significantly upregulated in the miR-30a-5p group, suggesting that autophagy was inhibited by miR-30a-5p regulating Beclin-1 (Fig. 3a, b). While the miR-30a-5p inhibitor increased Beclin-1 protein level and decreased p62 expression. An increaed ratio of LC3-II/LC3-I was observed for miR-30a-5p inhibitor-treated group compared to inhibitor-NC group, indicating that autophagy activity was promoted (Fig. 3c, d). Specific viral proteins with UL41 antibody indicated the process of viral infection. These results revealed that the miR-30a-5p inhibited autophagy by regulating Beclin-1in DEV-infected cells.

### miR-30a-5p promotes DEV-induced DEF cell apoptosis

**Fig. 4:**
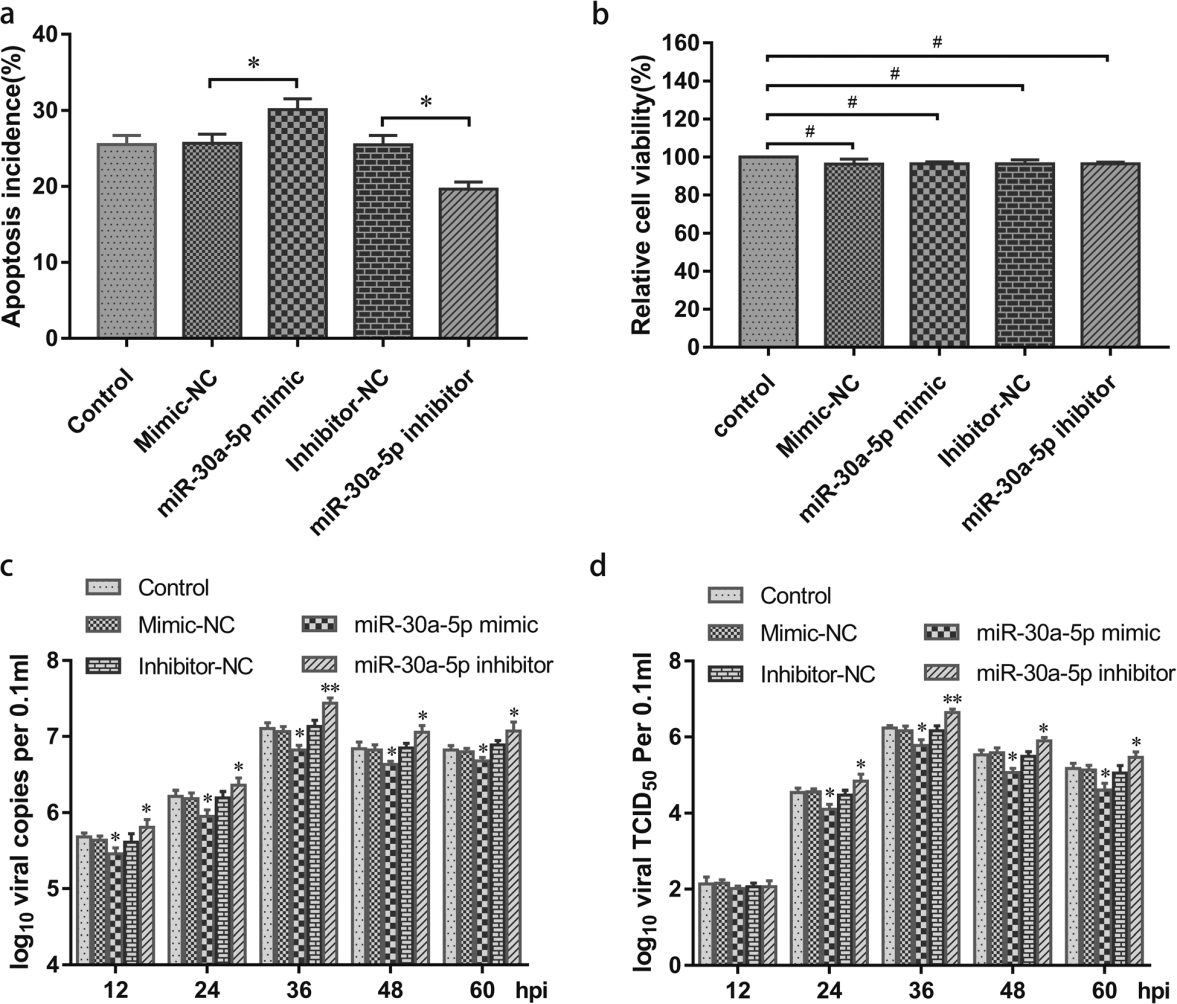
miR-30a-5p overexpression reduces DEV replication in DEF cells. **a** DEF cells were pretreated with control (without), mimic-NC, miR-30a-5p mimic, inhibitor-NC and miR-30a-5p inhibitor for 4 h and then infected with DEV for 36 h. Apoptosis was analyzed by Annexin V-FITC/PI staining using flow cytometry. **b** Changes in DEF viability following treatment with control (without), mimic-NC, miR-30a-5p mimic, inhibitor-NC and miR-30a-5p inhibitor for 4 h and then cultured in DMEM at 37 °C. The cell viability were tested by MTT assay kit at 36 h. **c** DEF cells were pretreated with control (without), mimic-NC, miR-30a-5p mimic, inhibitor-NC and miR-30a-5p inhibitor for 4 h and then infected with DEV. Viral copies were determined at the indicated time points. **d** DEF cells were pretreated with control (without), mimic-NC, miR-30a-5p mimic, inhibitor-NC and miR-30a-5p inhibitor for 4 h and then infected with DEV, and viral yields (TCID_50_ per 0.1 ml) were determined at the indicated time points. The data are presented as the mean ± SD for three independent experiments. *p < 0.05; **p < 0.01, compared with the control group. “ # ” indicates no significant difference, *P* > 0.05

A FCM was used to detect DEV-induced DEF cell apoptosis after transfecting control, miR-30a-5p mimic, miR-NC, miR-30a-5p inhibitor and inhibitor-NC. The results revealed that the percentage of apoptotic DEF cells was significantly increased at 36 hpi in the miR-30a-5p mimic group. However, the transfection of miR-30a-5p inhibitor significantly decreased the percentage of apoptotic cells in DEV-infected cells (Fig. 4a). These results showed that miR-30a-5p promoted DEV-induced apoptosis of DEF cells.

### Cell viability unaffected by miRNAs treatment

The miRNAs might have influenced cell viability and affected our results. The effects on cell viability of the miRNAs used in this study were measured by MTT assays. The viability of treated cells was almost equal to that of control cells, so the miRNAs treatments did not affect DEF cell viability (Fig. 4b).

### miR-30a-5p overexpression reduces DEV replication

To identify the potential function of miR-30a-5p in the viral replication process, DEF cells were transfected with control, miR-30a-5p mimic, miR-NC, miR-30a-5p inhibitor and inhibitor-NC for 4 h and then infected with DEV (MOI = 1.0). The genome copy number of DEV was measured by qRT-PCR at the indicated time points, and overexpression of miR-30a-5p strongly suppressed DEV replication at 12, 24, 36, 48 and 60 hpi, while the miR-30a-5p inhibitor significantly triggered DEV replication at the same time points (Fig. 4c).

Viral yields were determined by TCID_50_ at the indicated time points, and titers of progeny viruses collected from miR-30a-5p mimic group were lower than from control cells at 24, 36, 48 and 60 hpi. While viral titers in miR-30a-5p inhibitor group were significantly upregulated compared with the control group (Fig. 4d).

## Discussion

There have been several reports about miR-30a-5p target genes and their involvement in pathophysiological processes. For example, miR-30a-5p promotes the replication of porcine circovirus type 2 through enhancing autophagy by targeting 14–3-3 [15]. MiR-30a-5p downregulation contributes to the chemoresistance of osteosarcoma cells by activating Beclin-1-mediated autophagy [35]. The expression of miR-30a-5p is significantly downregulated in human colorectal cancer (CRC) tissues and CRC cell lines and may be a potential candidate target for CRC therapy [36]. Low expression of miR-30a-5p induces the proliferation and invasion of oral cancer by promoting the expression of FAP (*Homo sapiens* fibroblast activation protein α), and miR-30a-5p might be a new therapeutic target for oral cancer treatment [37]. In our previous study, miRNA expression profiles of virus and host were determined and analyzed in virulent DEV-infected DEF cells. The expression level of miRNA-30a-5p was significantly downregulated during the whole process of DEV infection [31], and similar results were confirmed by stem-loop qRT-PCR in this study (Fig. 1b). We speculate that miR-30a-5p plays an important role in regulating host-virus interactions.

Autophagy is an essential pathway for cellular homeostasis. Many studies have confirmed that some viruses can induce cells manipulate autophagy to promote their survival and replication [38, 39]. Examples include hepatitis C virus [40], egg drop syndrome virus (EDSV) [41], avian reovirus [42] and influenza A virus [43]. Rrecent study demonstrated that autophagy induced by DEV infection positively promotes viral replication [21]. However, the regulating relationship of autophagy in DEV-infected DEF cells is still unclear. In this study, our data showed that the expression of miR-30a-5p was downregulated and that Beclin-1 was upregulated after CHv infection of DEF cells. The Beclin-1 gene is recognized as a critical regulatory gene during autophagosome formation and maturation [44] and plays important roles in the replication of some viruses. For example, the replication of these three viruses (NDV, CSFV and DEV) was inhibited by siRNA knockdown of Beclin-1 gene level, which is required for autophagy [13, 14, 21]. Previous studies have demonstrated that miR-30a-5p regulates autophagy activity by targeting Beclin-1 mRNA [45–47]. Therefore, we speculated that there is the negative regulation relationship between Beclin-1 and endogenous miR-30a-5p in DEV-infected DEF cells. To confirm the regulation of miR-30a-5p on Beclin-1, bioinformatics analysis was performed using RNAhybrid software [31]. The results showed that miR-30a-5p was predicted to target the 3′-UTR region of Beclin-1. DLRA confirmed that overexpression of miR-30a-5p markedly reduced the luciferase level from Beclin-1 (Fig. 2a, b).

Western blot analysis demonstrated that overexpression of miR-30a-5p decreased decreased Beclin-1 protein level and the ratio of of LC3-II/LC3-I, enhanced the p62 protein level, which are related to autophagy. The expression of UL41 protein decreased (Fig. 3a, b). Whereas miR-30a-5p inhibitor attenuated DEV-induced cell autophagy and reversed the effect of miR-30a-5p. Both Beclin-1 and the ratio of LC3-II/LC3-I significantly increased in miR-30a-5p inhibitor group. The p62 protein level decreased and the expression of UL41 protein increased (Fig. 3c, d). The TCID_50_ and the viral copy test confirmed that DEV titers were significantly decreased in the miR-30a-5p mimic group compared with control group. Whereas transfection of miR-30a-5p inhibitor promoted DEV replication during the whole process of DEV infection (Fig. 4c and d). Our results were consistent with a previous report on autophagy induced by DEV [21], suggesting a key role for the miR-30a-5p/autophagy loop in DEV infection. These results strongly suggested that overexpression of miR-30a-5p decreased DEV replication by suppressing Beclin-1-mediated autophagy. Therefore, it is reasonable to conclude that downregulation of miR-30a-5p contributes to DEV replication by upregulating Beclin-1-mediated autophagy.

Apoptosis regulates embryonic development, cell turnover, and the immune response against tumor or virus-infected cells [48, 49]. Virus-induced cell apoptosis is involved in the pathogenesis of many viral infections [50, 51]. Our laboratory has discovered that DEV can induce apoptosis in the thymus, spleen and pancreatic lymphocytes of adult ducks and can cause apoptosis in DEFs in vitro [52, 53], and further confirmed that the mRNA levels and enzymatic activities of caspase-3, caspase-7 and caspase-9 were significantly increased during DEV-induced cell apoptosis [53]. Recent study has also reported miR-30a-5p can promote doxorubicin-induced osteosarcoma cell apoptosis by increasing the expression of cleaved caspase-3, and further certified that miR-30a-5p promotes chemotherapy-induced osteosarcoma cell apoptosis via repressing Beclin-1-mediated osteosarcoma autophagy [35]. In our study, flow cytometry demonstrated that overexpression of miR-30a-5p enhanced DEV-induced cell apoptosis (Fig. 4a). Nevertheless, miR-30a-5p inhibitor attenuated DEV-induced cell apoptosis and reversed the effect of miR-30a-5p (Fig. 4a). However, whehter or not the miR-30a-5p increased DEV-induced cell apoptosis by suppressing Beclin-1-mediated autophagy requires further confirmation.

## Conclusions

The results of this study showed that the miR-30a-5p/autophagy loop plays an important role in DEV infection, and this is the first report of miR-30a-5p in herpesvirus-induced autophagy. Our work confirms the existence of a novel regulatory pathway controlled by miR-30a-5p and its direct target, Beclin-1, that regulates DEV replication in DEFs. Therefore, miR-30a-5p and its target gene pathway may represent new treatment methods for duck viral enteritis disease.

## Data Availability

The datasets used or analysed during this study are included within the article.

## Abbreviations

CHv: Chinese virulent
CSFV: Classical swine fever virus
DEV: Duck enteritis virus
EDSV: Egg drop syndrome virus
IRS: Inverted repeated sequences
NDV: Newcastle disease virus
PCV2: Porcine circovirus type 2
PRRSV: Porcine reproductive and respiratory syndrome virus
qRT-PCR: Quantitative real-time reverse transcriotion PCR
TRS: terminal repeated sequences
UL: Unique long region
US: Unique short region
VZV: Varicella-zoster virus
